# Relationships between Dietary Patterns and Indices of Arterial Stiffness and Central Arterial Wave Reflection in 9–11-Year-Old Children

**DOI:** 10.3390/children7060066

**Published:** 2020-06-25

**Authors:** Pouya Saeedi, Jillian Haszard, Lee Stoner, Sheila Skeaff, Katherine E. Black, Brittany Davison, Harriet Harrex, Kim Meredith-Jones, Robin Quigg, Jyh Eiin Wong, Paula M. L. Skidmore

**Affiliations:** 1 Department of Human Nutrition, University of Otago, Dunedin 9054, New Zealand; pouya.saeedi@gmail.com (P.S.); jill.haszard@otago.ac.nz (J.H.); sheila.skeaff@otago.ac.nz (S.S.); katherine.black@otago.ac.nz (K.E.B.); brittany_davison@hotmail.com (B.D.); harriet.harrex@outlook.com (H.H.); 2 Department of Exercise and Sports Science, University of North Carolina, Chapel Hill, NC 27519, USA; stonerl@email.unc.edu; 3 Department of Medicine, University of Otago, Dunedin 9054, New Zealand; kim.meredith-jones@otago.ac.nz; 4 Cancer Society Social and Behavioural Research Unit, Department of Preventive and Social Medicine, Dunedin School of Medicine, University of Otago, Dunedin 9054, New Zealand; robin.quigg@otago.ac.nz; 5 Centre for Community Health Studies, Faculty of Health Sciences, Universiti Kebangsaan Malaysia, Kuala Lumpur 50300, Malaysia; wjeiin@ukm.edu.my

**Keywords:** arterial stiffness, augmentation index, children, dietary patterns, principal component analysis, pulse wave velocity

## Abstract

Arterial stiffness is an important marker of vascular damage and a strong predictor of cardiovascular diseases (CVD). Given that pathophysiological processes leading to an increased arterial stiffness begin during childhood, the aim of this clustered observational study was to determine the relationship between modifiable factors including dietary patterns and indices of aortic arterial stiffness and wave reflection in 9–11-year-old children. Data collection was conducted between April and December 2015 in 17 primary schools in Dunedin, New Zealand. Dietary data were collected using a previously validated food frequency questionnaire and identified using principal component analysis method. Arterial stiffness (carotid-femoral pulse wave velocity, PWV) and central arterial wave reflection (augmentation index, AIx) were measured using the SphygmoCor XCEL system (Atcor Medical, Sydney, Australia). Complete data for PWV and AIx analyses were available for 389 and 337 children, respectively. The mean age of children was 9.7 ± 0.7 years, 49.0% were girls and 76.0% were classified as “normal weight”. The two identified dietary patterns were “Snacks” and “Fruit and Vegetables”. Mean PWV and AIx were 5.8 ± 0.8 m/s and −2.1 ± 14.1%, respectively. There were no clinically meaningful relationships between the identified dietary pattern scores and either PWV or AIx in 9–11-year-old children.

## 1. Introduction

Non-communicable diseases (NCDs) such as cardiovascular diseases (CVD), respiratory diseases and diabetes are estimated to account for 89% of all deaths in New Zealand. CVD are the most prevalent NCDs in New Zealand accounting for 31% of the annual deaths across all age groups, while other NCDs such as chronic respiratory diseases and diabetes account for only seven and three percent of the total mortality, respectively [1].

CVD usually manifests during the middle age [2]. However, the pathophysiological processes underlying CVD begin during the first decade of life [3]. Early pathophysiological processes include the changes to functional and structural characteristics of arteries, resulting in an increased aortic arterial stiffness measured as pulse wave velocity (PWV) and changes in indices of wave reflection, especially augmentation index (AIx) [4,5].

At least in adults, both PWV and AIx are associated with lifestyle behaviours, including dietary intake [6,7,8,9]. The human diet is a combination of a diverse range of food items and nutrients, and arguably [10], dietary patterns are more likely to contribute to PWV and AIx than single foods or nutrients. Indeed, in epidemiological and interventional studies of adults, healthier dietary patterns such as vegetable-rich dietary patterns were associated with lower levels of markers of arterial stiffness and wave reflection [5,6,7]. 

Although studies in children have shown that CVD risk factors such as metabolic syndrome, diabetes and obesity contribute to increased risk of arterial stiffening [11], the relationship between lifestyle-related factors and arterial stiffness is not clear. Childhood is a period of life when lifestyle-related behaviours including dietary patterns are formed and fixed [12]. Given that pathophysiological processes underlying CVD begin in early childhood, and the same risk factors as in adulthood can be correlated with their progression, this study aimed to determine the association between dietary patterns and indices of aortic arterial stiffness (PWV) and arterial wave reflection (AIx) in a sample of 9–11-year-old children. Determining the association between modifiable risk factors and indices of arterial stiffness at early stages of life is fundamental, as these risk factors can have a significant impact on disease manifestation later in life [13].

## 2. Materials and Methods

This study is reported following the STROBE guidelines [14]. Ethical approval for this study was obtained in March 2015 from the University of Otago Ethics Committee (Ref No. 14/227).

### 2.1. Study Design and Sample Description

**Figure 1:**
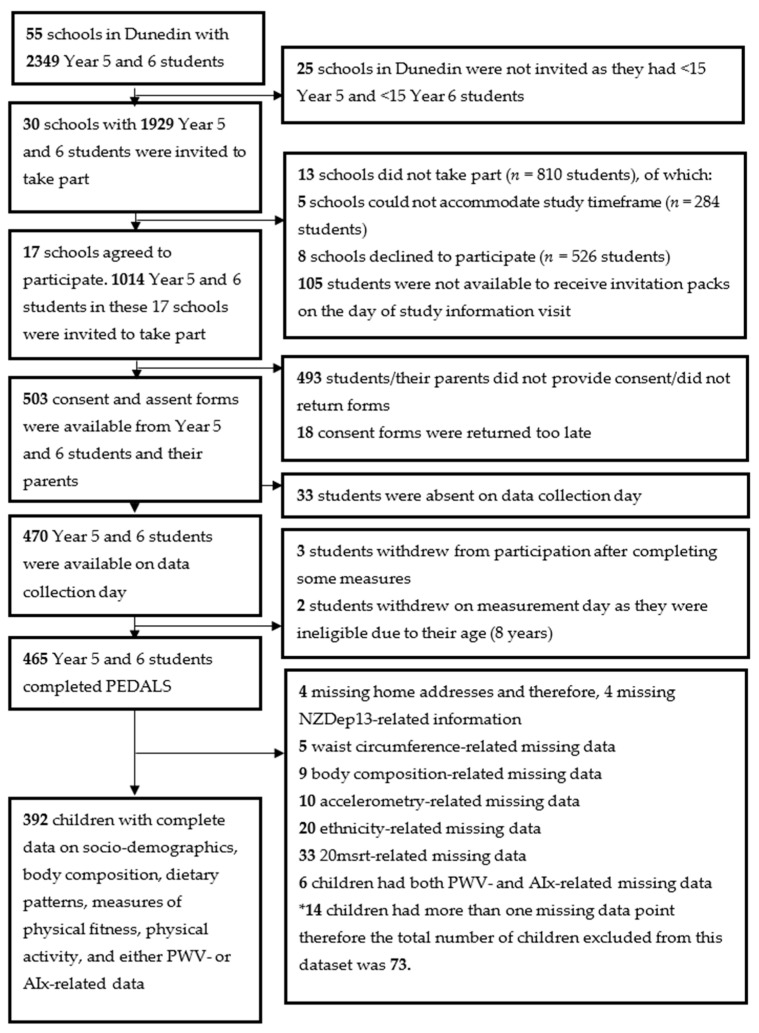
Study sample selection (schools and children) in the Physical activity, Exercise, Diet, And Lifestyle Study (PEDALS).

This study was part of a clustered observational study called “Physical activity, Exercise, Diet, And Lifestyle Study (PEDALS)”, carried out from April to December 2015 in primary schools in the city of Dunedin, New Zealand [15]. Of 55 primary schools, 30 had more than 15 students in Year 5 and 6 (usually between 9 and 11 years old) and were invited to participate in PEDALS (Figure 1) [15]. All interested Year 5 and 6 students were then invited, and to be eligible to participate in PEDALS, both the child’s assent and parent’s consent had to be received before data collection [15]. Data collection was performed within the respective schools throughout the duration of a school day. All data were collected in the class setting, with arterial- and anthropometric-related measurements conducted in a private and temperature-controlled space. The cardiorespiratory fitness test took place on school grounds (asphalt surface). 

### 2.2. Sample Size Estimation

PEDALS was designed to determine the prevalence of lifestyle behaviours in school children aged 9–11 years. Sample size calculation has been explained in detail in a previously published study [15]. Briefly, clustered sampling was used, and schools were considered as the sampling units. With this, “a sample size of 357 participants would be required to determine prevalence to a precision of at least ±9.0%”. Furthermore, at least 400 children were required, taking into account 10% incomplete data. The sample size would also be large enough to examine relationships between continuous variables using appropriate regression models. 

### 2.3. Study Variables

PWV and AIx were treated as dependent variables and measured using the SphygmoCor XCEL (Atcor Medical, Sydney, Australia) system, while principal component analysis (PCA) derived dietary patterns were the independent variables (explained in more details in the following sections). 

#### 2.3.1. Dietary Intake and Dietary Pattern Derivation

Frequency of food consumption was assessed using a previously validated 28-item non-quantitative food frequency questionnaire (PEDALS-FFQ) [16]. Using information from the PEDALS-FFQ and based on the principal component analysis (PCA) with varimax orthogonal rotation, dietary patterns were identified. The number of dietary patterns to be retained was determined based on eigenvalues > 1, identification of the elbow in the scree plot, and the interpretability of factors within components [17]. For comparability and interpretability of the results, food items/groups with absolute factor loadings ≥ 0.2 were considered as the most strongly correlated food items/groups within each component and were therefore used to identify the dietary patterns. Overall, two dietary patterns (i.e., “Snacks” and “Fruit and Vegetables”) were clearly identifiable, and none of the food items/groups were cross-loaded. The development of the dietary patterns has been reported in further detail elsewhere [18]. 

#### 2.3.2. Arterial Stiffness (PWV) and Wave Reflection (AIx) 

The SphygmoCor XCEL system was used to assess PWV and AIx. It is a common practice to ask participants to abstain from eating large meals and drinking prior to arterial-related measurements [19]. However, as our data collection was carried out throughout one school day, it was not practical to ask children to stay fasted during the day and thus measurements were conducted in a non-fasting state. Before measurements were taken, a trained researcher explained the procedures to the children to provide comfort and reduce tension. All measurements were conducted in a quiet and private space, with children resting in the supine position for at least five minutes prior to testing to provide haemodynamic stability [19]. 

To measure AIx, an appropriately sized brachial cuff was positioned on the left upper arm and used to capture brachial pressure waveforms. Subsequently, a central waveform was generated using a validated generalized transfer function to calculate AIx [20]. AIx is calculated as augmentation pressure (AP) relative to central pulse pressure, where AP is the difference between the second (generated by the reflected waves) and first systolic peaks (generated by left ventricular ejection) [20]. Overall, 55 children were excluded following expert review (LS) of the waveform.

PWV was measured only once and immediately following the AIx measurement, by simultaneous recording of proximal (carotid) and distal (superficial femoral) pulse pressure waveforms using a tonometer and volume displacement cuff (SC10, Hokanson, Bellevue, WA, USA), respectively. PWV is calculated by dividing pulse transit time (PTT) by PWV distance (arterial path length). The path length was determined as the direct distance (carotid to femoral), corrected for the femoral segment between the femoral artery (groin region) and the top edge of the femoral cuff. The direct distance measurement method (carotid to femoral) has been the most commonly used method in prognostic and survival studies and is a standard method for daily practices [21]. PTT was calculated by the XCEL as the time between the diastolic feet of the tonometer (carotid) and cuff (femoral) arterial waveform recordings. All measurements were checked for the quality of waveforms and were repeated if necessary. An expert (LS) also reviewed and screened all the waveforms. 

### 2.4. Study Covariates

Study covariates (i.e., socio-demographic factors, anthropometric factors, body composition, cardiorespiratory fitness, moderate-vigorous physical activity, heart rate and mean arterial pressure) were assessed using standardized protocols, explained in the following sections. 

#### 2.4.1. Socio-Demographic, Anthropometric Factors and Body Composition

Child ethnicity, determined by their parents, was categorised into two groups: Māori (indigenous population of New Zealand) and non-Māori. Socio-economic status was determined using the 10-point NZDep13, the New Zealand socio-economic deprivation index [22]. NZDep13 scores were grouped as follows: low deprivation (scores 1–3), middle deprivation (scores 4–7) and high deprivation (scores 8–10). 

Children’s height was measured to the nearest 0.1 cm, using a portable stadiometer (WSHRP, Wedderburn®, Dunedin, New Zealand), with children’s head in the Frankfort Plane position. Two measurements were obtained, and a third measurement was taken if the first two readings differed by more than 0.5 cm. Weight and body composition were measured by a calibrated bioelectrical impedance analyser (BIA) (TBF-300A, Tanita, Japan) [15]. 

Fat mass index (FMI, kg/m^2^) was calculated as fat mass (kg) divided by height in meters squared (m^2^), while fat-free mass index (FFMI, kg/m^2^) was determined by dividing fat-free mass (kg) by height (m^2^) [15]. Children’s weight status (underweight, normal weight, overweight and obese) was assessed using the International Obesity Task Force (IOTF) body mass index (BMI) cut-offs [23]. Mean arterial pressure and heart rate were measured using the SphygmoCor XCEL system.

#### 2.4.2. Cardiorespiratory Fitness 

Cardiorespiratory fitness was measured using the 20-meter shuttle run test (20msrt). Details on the 20msrt and its assessment are described elsewhere [15]. The Léger equation, which is a reproducible (r = 0.9) and valid (r = 0.7) measure in 8–19-year-old populations was used to predict relative maximal oxygen uptake (V_O2max_, ml/kg/min) and consequently the cardiorespiratory fitness level of children [24]. 

#### 2.4.3. Physical Activity 

Physical activity was measured objectively using a wrist-worn accelerometer (Actigraph GT3X+, Pensacola, FL, USA). Detailed information has been reported elsewhere [15]. Briefly, children were asked to wear the accelerometer at all times for eight consecutive days on their non-dominant wrist, except while swimming, showering, or playing water sports and were initialised using five second epochs. A valid day was defined as at least eight hours of wear time, and children who had less than three valid days were removed from data analyses. A non-wear time was considered as 20 minutes of consecutive zero counts, only during the awake periods [25]. To assess moderate-vigorous physical activity of children in PEDALS, the cut-points developed by Chandler et al for 8-12-year-old children were used [26]. Detailed information on data cleaning and scoring of physical activity data are presented elsewhere [27]. Other covariates including heart rate and mean arterial pressure were using the SphygmoCor XCEL system.

### 2.5. Data Handling and Statistical Analyses 

All statistical analyses were performed in Stata (version 12.1, StataCorp, College Station, TX, USA). Continuous variables were presented as either geometric means (95% CI) or means and standard deviations (SD) if data were normally distributed. Categorical variables are shown as numbers and percentages. For questionnaire data, imputation was applied if missing data were part of a set of questions with at least four sub-questions. Data from the PEDALS-FFQ were the only imputed data in this study, using a worst-case scenario if 75.0% of the questions from each set of questions had been completed. 

Mixed effects regression models (school as a random effect) were used to investigate relationships between dietary pattern scores and indices of arterial stiffness and wave reflection. Three models were used, adjusted for important covariates based on the findings from previous research and the bivariable models. Model 1 was treated as an unadjusted model. Model 2 was adjusted for age, sex, ethnicity and NZDep13, while Model 3 was further adjusted for heart rate, mean arterial pressure, moderate-vigorous physical activity, cardiorespiratory fitness, FMI and FFMI. There were multiple measures of body composition including BMI and BIA-related measures such as FMI and FFMI. As FMI and FFMI represent different aspects of body composition (compartmentalized body composition), these two measures were included in the multivariable models rather than BMI. Interaction terms between sex and dietary patterns were included in regression models. As there was no significant interaction (*p* < 0.05), stratified analyses are not presented. Regression coefficients (β) with 95% CI are presented, which estimate mean differences in PWV (m/s) or AIx (%) for a SD higher dietary pattern score. 

## 3. Results

### 3.1. Participants

Seventeen schools (56.7% of all invited schools) with 1014 eligible Year 5 and 6 children agreed to take part in PEDALS (Figure 1). Overall, 503 consent and assent forms were received prior to data collection. Of 503 children, 465 participated in the study and 392 had complete data on socio-demographic factors, measures of body composition, physical fitness, physical activity and either PWV or AIx (Figure 1). 

Of the 392 children, three had PWV-related missing data, either because they refused to be examined or because carotid pulse waves could not be detected. Data on the remaining 389 children was used to perform PWV-related analyses, while AIx-related analyses were based on data from 337 children due to the quality of the waveforms (Table 1). 

Only data from children with all relevant variables were included in data analyses. Comparing children who were included in AIx-related analyses and those who were excluded, no significant differences were found in relation to socio-demographic characteristics, anthropometric (FMI, FFMI and BMI Z-score) and lifestyle factors (moderate-vigorous physical activity and dietary pattern scores) (all *p* > 0.05) (data not shown). However, children who were excluded from AIx-related analyses had significantly higher heart rate (82.0 ± 19.0 vs. 77.0 ± 10.0 bpm; *p* = 0.002) and mean arterial pressure (86.0 ± 12.0 vs. 82.0 ± 7.0 mmHg; *p* = 0.004) than those who were included in the analyses (data not shown).

### 3.2. Characteristics of Participants

General characteristics of the sample population are presented in Table 1. Based on the NZDep13 standards, the majority (82%) of participants had middle or high socio-economic status. About 88.0% of children identified as non-Māori, and 76% had normal weight status. 

### 3.3. Dietary Patterns Identification

Two dietary patterns, accounting for 37.9% of the total variation, were identified: (I) “Snacks”, highly and positively loaded for salty snacks, sweet baked snacks (e.g., fruit pies), lollies (i.e., confectioneries or candies), sweet snacks (e.g., chocolate bars), non-dairy drinks, ice cream, spreads, pasta and white bread; (II) “Fruit and Vegetables”, highly and positively loaded for fruit, vegetables, dairy products, breakfast cereals, brown bread, other meat and processed meat. Detailed information can be found elsewhere [18]. 

### 3.4. Bivariable Analyses

The physiological, anthropometric and lifestyle characteristics of children with PWV or AIx data are presented in Table 2. Mean PWV and AIx were 5.8 ± 0.8 m/s and −2.1 ± 14.1%, respectively. Normalised AIx to heart rate of 75 bpm was −1.4 ± 14.9%. Based on the bivariable models, FMI (β = 0.1, 95% CI = 0.0, 0.1 m/s) and BMI Z-score (β = 0.1, 95% CI = 0.1, 0.2 m/s) were significantly positively associated with PWV. Moreover, heart rate and mean arterial pressure were significantly associated with a 0.01 m/s (95% CI = 0.00, 0.02 m/s) and 0.03 m/s (95% CI = 0.02, 0.04 m/s) higher PWV, respectively. 

FFMI was significantly associated with AIx; for every kg/m^2^ higher FFMI, there was a 1.6% (95% CI = −2.7, −0.5%) lower AIx. Furthermore, each h/d of moderate-vigorous physical activity and each ml/kg/min higher V_O2max_ were significantly associated with 0.5% (95% CI = −0.8, −0.1%) and 0.4% (95% CI = −0.7, −0.1%) lower AIx, respectively. Mean arterial pressure was positively associated with AIx, as such every mmHg higher mean arterial pressure was associated with a 0.4% (95% CI = 0.2, 0.6%) higher AIx (data not shown).

### 3.5. Association between Dietary Pattern Scores and PWV and AIx

There was no interaction between dietary patterns and sex, and thus, stratified analyses are not presented. The relationships between dietary pattern scores and PWV and AIx are presented in Table 3. There were no significant relationships between PWV and dietary pattern scores (i.e., “Snacks” and “Fruit and Vegetables”) in either the bivariable model (Model 1) or multivariable models (Models 2 and 3). Similarly, we did not find significant associations between dietary pattern scores and AIx in any of the models. 

## 4. Discussion

PEDALS examined the relationships between dietary patterns and indices of arterial stiffness (PWV) and arterial wave reflection (AIx) in a sample of exclusively primary school aged children. No clinically significant relationships were found between dietary pattern scores and PWV and AIx in this population. 

Overall, there are limited information available on the relationship between dietary patterns and arterial health in children [28]. Although dietary patterns are reported to be associated with PWV and AIx in a high-risk population of children and adolescents (obese, high blood pressure, diabetic) [29,30], there was no such evidence in our dominantly normal weight children. Similar to our findings, dietary patterns (healthy and unhealthy) were not associated with PWV in a sample of 7–10-year-old Italian children [31]. The absence of the association between dietary pattern scores and PWV and AIx in the current study could be due to the fact that changes to functional and structural characteristics of arteries in asymptomatic populations are a slow process. Changes to arteries, although begin during the first decade of life [3], might not, however, be detectable at this early stage of life in a sample of apparently healthy children. 

Eating habits are an important correlate of childhood obesity [32,33]. Evidence from previous nutrition surveys in New Zealand shows that younger children have healthier diets than older children [34,35]. A possible explanation for healthier habits in younger children than their older counterparts can be more parental influence over younger children’s behaviour. Similarly, in our study, the majority of the children reported healthy eating behaviours (i.e., frequent consumption of fruits, vegetables and dairy products) [15].

Other available studies were undertaken in populations, the majority of whom were adolescents with already established chronic conditions [29,30]. Lamichhane et al. [29] found that a dietary pattern characterised by high consumption of sweetened beverages, diet soda, eggs and high-fat meats was unfavourably associated with AIx in 10–19-year-old Americans. Lydakis et al. [30] also reported a significant inverse association between the degree of adherence to the Mediterranean diet (e.g., high consumption of fruits, vegetables, olive oil, cereals, legumes and nuts, moderate intake of fish and dairy products and low amounts of meat and meat products) and AIx in 12–15-year-old Greek adolescents. Overall, the small number of studies and differences in population limit the comparison. Inconsistencies in findings between our study and previous research may be attributed in part to differences in health status of the sample populations. Unlike our cohort who were 9–11-year-old healthy children with the majority (76%) classified as normal weight, the study by Lamichhane et al. and Lydakis et al. included adolescents with type 1 diabetes and high prevalence of overweight and obesity (43.3%), respectively. 

Presence of traditional CVD risk factors (e.g., high BP, central obesity, dyslipidaemia and inflammatory markers) and endothelial dysfunction in individuals with type 1 diabetes are considered as important correlates of indices of arterial stiffness (e.g., AIx) [36,37,38]. In addition, an adverse relationship between less healthy eating behaviours, and endothelial function has been found in individuals with type 1 diabetes [39]. Therefore, a high-risk population (diabetic) with unhealthy eating habits (e.g., consumption of sweetened beverages, diet soda and high-fat meat) may have been more likely to develop arterial-related complications (AIx) than our apparently healthy cohort, who were mainly normal weight. Furthermore, obesity has been documented as a predisposing condition, which increases arterial stiffness [40]. Evidence shows that the presence of CVD risk factors in obese populations may also affect their endothelial function and therefore properties of wave reflection such as AIx [41,42]. Thus, in overweight and obese adolescents, the arterial stiffening process may have been started to a great extent compared with their normal weight peers. Having said that, in the absence of a high prevalence of obesity in our population, detrimental stiffening process may not have been started. 

Guidelines are available to tackle childhood obesity, through identifying at-risk children based on their BMI and thus help them to slow down the weight gain process. Concerning the growing obesogenic environment, there is, however, a need for specific guidelines for healthy-weight children to control their current weight status and prevent the accumulation of excess body fat as they grow older and, therefore, to prevent obesity-related complications.

The main strengths of our study include a relatively large sample of children, objective assessment of physical fitness (i.e., cardiorespiratory fitness) and physical activity, as well as a comprehensive assessment of indices of arterial stiffness considering a wide range of confounders including socio-economic, lifestyle and physiological factors. The SphygmoCor XCEL system was used to assess arterial health in the PEDALS population non-invasively. This system uses a more convenient and less intrusive approach than the older SphygmoCor systems to assess arterial stiffness, which has made it feasible to obtain arterial health-related measurements from a large number of children in a non-clinical setting.

We had a cohort of healthy children in PEDALS with the majority categorised as being of normal body weight. Thus, generalising our findings to a high-risk population (e.g., obese children or children with diabetes) should be performed with caution, as a high-risk population has a higher chance of developing a progressive vascular dysfunction than those with a normal body weight. Furthermore, although a validated FFQ was used to collect dietary data, identified dietary patterns might not have been based on children’s usual diet. Furthermore, our participants belonged to a limited age range (9–11 years old), and consequently, our results might not be generalisable to younger or older aged children.

Overall, there were no clinically significant relationships between the dietary pattern scores and indices of arterial stiffness and wave reflection in 9–11-year-old children, which may be attributed to the population health status, with majority of them having normal weight status.

## Figures and Tables

**Table 1:** General characteristics of the PEDALS population with PWV or AIx data.

Demographic Variable ^1^	Children with PWV Data *n* = 389	SD	Children with AIx Data *n* = 337	SD
Age (years)	9.7	0.7	9.7	0.7
Sex				
Boys	194 (49.9)	-	166 (49.3)	-
Girls	195 (50.1)	-	171 (50.7)	-
School year				
Year 5	222 (57.1)	-	195 (57.9)	-
Year 6	167 (42.9)	-	142 (42.1)	-
Ethnicity ^2^				
Māori	44 (11.3)	-	42 (12.5)	-
Non-Māori	345 (88.7)	-	295 (87.5)	-
NZDep13 ^3^				
Low deprivation	172 (44.2)	-	149 (44.2)	-
Middle deprivation	148 (38.1)	-	131 (38.9)	-
High deprivation	69 (17.7)	-	57 (16.9)	-
Weight status ^4^				
Underweight	20 (5.1)	-	19 (5.6)	-
Normal weight	296 (76.1)	-	257 (76.2)	-
Overweight	55 (14.2)	-	45 (13.4)	-
Obese	18 (4.6)	-	16 (4.8)	-

PWV: pulse wave velocity; SD: standard deviation; AIx: augmentation index. ^1^ Data are expressed as mean and SD for numerical variables and counts (percentages) for categorical variables. ^2^ Māori is the indigenous population of New Zealand. Non-Māori ethnic group includes New Zealand Europeans and Other ethnicities. ^3^ NZDep13, indicator of New Zealand socio-economic status. ^4^ Based on the International Obesity Task Force (IOTF) cut-offs [23].

**Table 2:** Physiological, anthropometric and lifestyle covariates in children with PWV or AIx data.

Variable ^1^	Children with PWV Data *n* = 389	SD	Children with AIx Data *n* = 337	SD
PWV (m/s)	5.8	0.8	-	-
AIx (%)	-	-	−2.1	14.1
AIx@HR75 ^2^ (%)	-	-	−1.4	14.9
PP	43.0	6.5	42.8	6.3
cPP	26.5	4.5	26.4	4.3
PP/cPP ratio	1.6	0.1	1.6	0.1
Heart rate (bpm)	74.6	10.0	76.7	9.7
Mean arterial pressure (mmHg)	82.8	8.1	82.4	7.3
V_O2max_ (ml/kg/min)	48.8	4.8	48.9	4.8
FMI (kg/m^2^)	3.4 (3.2, 3.5)	-	3.3 (3.2, 3.5)	-
FFMI (kg/m^2^)	14.1	1.2	14.1	1.1
BMI Z-score ^3^	0.4	1.1	0.4	1.0
MVPA (h/d)	2.2 (2.0, 2.4)	-	2.2 (2.0, 2.5)	-
Snacks score	−0.0	1.0	−0.0	1.0
Fruit and Vegetables score	0.0	1.0	0.0	1.0

PWV: pulse wave velocity; AIx: augmentation index; V_O2max_: maximal oxygen uptake; FMI: fat mass index; FFMI: fat-free mass index; BMI: body mass index; MVPA: moderate-vigorous physical activity; PP: pulse pressure; cPP: central pulse pressure; SD: standard deviation. ^1^ Continuous variables with skewed distribution (FMI, MVPA) are presented as geometric mean (95% CI). Normally distributed continuous variables are presented as mean and SD. ^2^ Normalised AIx to heart rate of 75 bpm. ^3^ Based on WHO Z-scores.

**Table 3:** Association of dietary pattern scores with PWV (*n* = 389) and AIx (*n* = 337).

Variable ^1^	Snacks Score			Fruit and Vegetables Score		
	β ^2^	95% CI	*p* Value	β ^2^	95% CI	*p* Value
**PWV (m/s)**						
Model 1	0.02	−0.05, 0.09	0.61	−0.00	−0.09, 0.08	0.93
Model 2	0.00	−0.06, 0.06	0.97	−0.01	−0.10, 0.08	0.77
Model 3	−0.02	−0.08, 0.05	0.61	−0.05	−0.13, 0.04	0.31
**AIx (%)**						
Model 1	−0.05	−2.00, 1.91	0.96	−0.85	−1.77, 0.08	0.07
Model 2	0.46	−1.46, 2.39	0.64	−0.57	−1.50, 0.35	0.23
Model 3	0.32	−1.28, 1.93	0.70	−0.37	−1.26, 0.52	0.41

PWV, pulse wave velocity; AIx, augmentation index; CI, confidence interval. ^1^ To assess the relationship between dietary pattern scores and indices of arterial stiffness and wave reflection, mixed effects linear regression was used with school as a random effect, adjusted for covariates as described by models 1 to 3: Model 1, unadjusted; Model 2, adjusted for age, sex, ethnicity, NZDep13; Model 3, adjusted for covariates in Model 2 + heart rate, mean arterial pressure, moderate-vigorous physical activity, V_O2max_, fat mass index (FMI) and fat-free mass index (FFMI). ^2^ β represents the mean difference in PWV (m/s) or AIx (%) associated with a SD higher in dietary pattern score.
